# Biological Characteristics and Patterns of Codon Usage Evolution for the African Genotype Zika Virus

**DOI:** 10.3390/v12111306

**Published:** 2020-11-14

**Authors:** Martin Faye, Naimah Zein, Cheikh Loucoubar, Manfred Weidmann, Ousmane Faye, Marielton dos Passos Cunha, Paolo Marinho de Andrade Zanotto, Amadou Alpha Sall, Oumar Faye

**Affiliations:** 1 Virology Department Institut Pasteur of Dakar, Dakar 220, Senegal; martin.faye@pasteur.sn (M.F.); ousmane.faye@pasteur.sn (O.F.); Amadou.SALL@pasteur.sn (A.A.S.); 2 Institute of Genetics and Molecular and Cellular Biology, CEDEX, 10142/67404 Illkirch, France; Naimah.zein@hotmail.fr; 3 Bioinformatics, Biostatistics and Modeling group, Institut Pasteur of Dakar, Dakar 220, Senegal; Cheikh.loucoubar@pasteur.sn; 4 Institute of Aquaculture University of Stirling, Stirling FK9 4LA, Scotland, UK; m.w.weidmann@stir.ac.uk; 5 Microbiology Department, Institute of Biomedical Sciences, University of São Paulo (USP), São Paulo, SP 05508020, Brazil; marieltondospassos@gmail.com (M.d.P.C.); pzanotto@usp.br (P.M.d.A.Z.)

**Keywords:** African genotype Zika virus, codon adaption dynamics, biological characteristics

## Abstract

We investigated temporal trends of codon usage changes for different host species to determine their importance in Zika virus (ZIKV) evolution. Viral spillover resulting from the potential of codon adaptation to host genome was also assessed for the African genotype ZIKV in comparison to the Asian genotype. To improve our understanding on its zoonotic maintenance, we evaluated in vitro the biological properties of the African genotype ZIKV in vertebrate and mosquito cell lines. Analyses were performed in comparison to Yellow fever virus (YFV). Despite significantly lower codon adaptation index trends than YFV, ZIKV showed evident codon adaptation to vertebrate hosts, particularly for the green African monkey *Chlorocebus aethiops*. PCA and CAI analyses at the individual ZIKV gene level for both human and *Aedes aegypti* indicated a clear distinction between the two genotypes. African ZIKV isolates showed higher virulence in mosquito cells than in vertebrate cells. Their higher replication in mosquito cells than African YFV confirmed the role of mosquitoes in the natural maintenance of the African genotype ZIKV. An analysis of individual strain growth characteristics indicated that the widely used reference strain MR766 replicates poorly in comparison to African ZIKV isolates. The recombinant African Zika virus strain ArD128000*^E/NS5^ may be a good model to include in studies on the mechanism of host tropism, as it cannot replicate in the tested vertebrate cell line.

## 1. Introduction

Zika virus (ZIKV) is a mosquito-borne *Flavivirus* belonging to the *Flaviviridae* family and the Spondweni serocomplex. ZIKV was first isolated in 1947 in Uganda from a febrile sentinel rhesus monkey during a Yellow fever virus (YFV) study [1]. Its natural transmission cycle involves mainly mosquitoes of the *Aedes* genus and monkeys [2,3,4,5,6], while human infections are accidental and generally asymptomatic. However, in clinical pictures of human infection, the Zika fever (ZF) ranges from a febrile syndrome associated with fever, headache, arthralgia, myalgia, conjunctivitis and cutaneous rash to severe neurological symptoms such as Guillain-Barré syndrome and microcephaly in newborns [7,8,9,10]. Overall, sexual intercourse, perinatal infection and blood transfusion have been reported as potential routes of direct transmission of ZIKV among humans [11,12,13].

The ZIKV genome consists of a linear, single-stranded, positive sense RNA of 10.794 kilobases (Kb) in length. Like other members of the *flavivirus* genus, the polyprotein of ZIKV is flanked by noncoding regions (5′ and 3′NCR) and encodes for three structural proteins (5′-C-prM-E-3′) and seven nonstructural proteins (5′-NS1-NS2A-NS2B-NS3-NS4A-NS4B-NS5-3′). The envelope (E) and the RNA polymerase (NS5) are major viral proteins mediating binding and membrane fusion and viral replication, respectively [14,15,16]. ZIKV is associated with a large genetic diversity clustered in three lineages (East African, West African and Asian), and the ZIKV genome has been shown to be subject to recombination and N-glycosylation in nature [17].

Despite serological evidence and entomological data demonstrating ZIKV circulation from 1950s to 1990s, ZIKV remained limited to a sylvatic cycle in equatorial regions of East and West Africa and South-East Asia, with few sporadic human cases [1,18,19,20,21,22,23,24,25,26]. A ZIKV outbreak in humans occurred in the Yap States of the Federal States of Micronesia in 2007 [27,28]. Subsequently, ZIKV infections were reported in Cambodia and French Polynesia in 2010 and 2013, respectively, and spread to seventy four countries worldwide [29,30]. Since 2013, ZIKV has shown a high prevalence in the Americas and was responsible of a large epidemic in 2015 with Brazil as the epicenter [30,31,32,33].

Since the replication cycle of viruses is solely dependent on host translational machinery, efficient viral replication in a host species of usually different genetic background requires a certain degree of genetic adaptation. Thus, mutational and selection pressures can result in adaptation to hosts [34,35]. The Codon Adaptation Index (CAI) is a measure of the synonymous codon usage bias, allowing comparisons of codon usage preferences in different organisms and assessing how well the codon preferences of a virus matches that of a reservoir host or vector [36,37,38].

Despite several entomological isolations and sporadic human cases reported in different countries in Africa [18,22,39,40,41], ZF outbreaks in humans have been mostly attributed to the Asian genotype [27,28,29,30,31,32,33]. This observation raises the question of whether the adaptation to humans of the African genotype differs from that of Asian genotype. The low public health impact reported for the African genotype ZIKV could be associated with its intrinsic properties. Investigation of the biological and genetic characteristics of African ZIKV strains could improve our understanding of their zoonotic maintenance. Here, we describe an analysis of the codon usage of the African genotype in comparison to the Asian genotype. We also analyze in vitro the biological properties of six ZIKV strains circulating in Africa in mosquito and vertebrate cells. Since ZIKV was first isolated during a Yellow fever disease investigation [1,21,40], and these two viruses share the main vector (*Aedes aegypti*) [2], all analyses and experiments were performed in comparison to YFV, which has a long history of infection in humans and is still a major public health problem, especially in West Africa, where it circulates almost every year [42].

## 2. Materials and Methods

### 2.1. Sequence Datasets Analyzed

Nucleotide coding sequences from isolates of ZIKV [*n* = 376 including sequences from African (*n* = 20) and Asian (*n* = 356) genotypes], YFV (*n* = 51) and tobacco mosaic virus (TMV) (*n* = 68) that had information on year and place of isolation and representing all data available to date on Genbank (www.ncbi.nlm.nih.gov/genbank/) were downloaded and used in this study. The complete coding sequences were aligned and curated using Muscle algorithm (http://www.drive5.com/muscle/) within the Unipro UGENE software (http://ugene.net/download.html) [43].

### 2.2. Codon Usage Tables

In order to calculate the CAI for the coding regions of the TMV, YFV and ZIKV viruses, we used codon usage tables provided by the CoCoPUTs platform [44,45] for vertebrate hosts including *Homo sapiens, Macaca mulatta, Erythrocebus patas* and *Chlorocebus aethiops*, and invertebrate vectors including *Aedes aegypti, Aedes albopictus* and *Culex pipiens* (Table 1).

### 2.3. Codon Usage Biases Analysis of Viral Coding Genes

We applied the calculation of the CAI value using a frequency table for vertebrate and invertebrate genes from the CoCoPUTs platform (Table 1) [44,45]. The CAI values were calculated to measure the synonymous codon usage bias using the CAIcal program [46]. To evaluate the statistical support of the CAI values, we defined a threshold value or expected CAI (e-CAI) [47] by generating random sequences with similar GC content, amino acid composition and sequence length to each query sequence (TMV, YFV and ZIKV). CAI values above the e-CAI were interpreted as statistically significant, meaning that codon similarity arose from codon preferences, rather than from internal biases [47].

### 2.4. Adaptation Levels to Host Genome

In order to assess the adaptation level of the African ZIKV genotype to invertebrate and vertebrates, codon usage of its sequences (*n* = 20) was then analyzed in comparison to the Asian genotype ZIKV (*n* = 356). Sequences of YFV (*n* = 51) and TMV (*n* = 68) were used as positive and negative controls, for adaptation and no adaptation to the human genome, respectively. We also did CAI analyses for each viral coding region using both *A. aegypti* and *H. sapiens* codon usage tables. Additionally, a principal component analysis to verify clustering patterns according to viral genotypes was performed considering the different genomic regions of the virus.

### 2.5. In Vitro Replication Kinetics of African Zika Virus Strains

#### 2.5.1. Samples

The samples used in this study were provided by the WHO Collaborating Centre for Arboviruses and Hemorrhagic Fevers Reference and Research located at the Institut Pasteur de Dakar in Senegal, and were obtained during a routine surveillance program for multiple mosquito-borne viruses conducted for over 50 years [48]. Strains with evidence of recombination and/or presenting motifs of N-glycosylation on the E protein were included in this study in order to assess the impact of these mechanisms on ZIKV African genotype growth (Table 2).

#### 2.5.2. Viral Stocks Preparation

*Aedes pseudoscutellaris* clone 61 (Ap61) continuous cell lines were inoculated with strains used in this study and incubated at 28 °C without CO_2_ until a cytopathic effect (CPE) was observable. Infection status was assessed by indirect immunofluorescence assay (IFA) as previously described [49], and virus titers were determined by plaque assay, as previously described [50], using porcine kidney stable cells (PS cells; American Type Culture Collection, Manassas, VA). The supernatants of infected cells were frozen at −80 °C and used as viral stocks for growth kinetics experiments.

#### 2.5.3. Growth Kinetics

An amount of 2.4 × 10^5^ Ap61 or African green monkey kidney epithelial cells (Vero cells; *Cercopithecus aethiops*; Sigma Aldrich, France) were seeded into each well in a volume of 400 µL of appropriate medium [51]. In addition, viral stocks were standardized to the number of plaques forming units per milliliter (PFU/mL), as in previous studies [42,51,52], and cells in each well were infected with 2.4 × 10^3^ plaque-forming units (PFU) of virus in 400 µL of medium, resulting in a multiplicity of infection (MOI) of 0.01. Experiments were conducted in 12-well plates using one plate per virus strain with one uninfected well as a negative control. Plates with infected Ap61 and Vero cells were incubated at 28 °C without CO_2_, and at 37 °C with CO_2_, respectively. After an incubation time of 4 h, the medium was collected and replaced with 2 mL of new medium to set a start point for the growth kinetics (T0).

At different time points corresponding to 22, 28, 50, 75, 99, 124, and 146 h post infection (hpi), one well per plate was harvested and the supernatants were collected and frozen at −80 °C in small aliquots until use. Cells were washed once with phosphate-buffered saline (PBS) and then collected in 500 µL PBS. A volume of 20 µL of cell suspension was dried on a glass slide for a subsequent immunofluorescence assay [50] to measure viral antigens production; the remaining cell suspensions were frozen at −80 °C. Then, RNA was extracted from cell fractions, and supernatants and copy numbers of the genome were quantified using a real-time reverse transcriptase quantitative polymerase chain reaction (RT-qPCR) and a standard equation previously described for ZIKV quantification [53]. Finally, infectious viral particles were measured in supernatants by plaque assay [50]. The growth kinetics experiments were performed three times on each cell type.

#### 2.5.4. Comparison with Data from In Vitro Growth of YFV

Data of genome production obtained on Ap61 cells during these experiments with ZIKV were also compared to those we previously obtained for YFV in the same conditions with the same MOI as for ZIKV [42] to better understand the zoonotic maintenance of ZIKV. Information about the YFV strains used in the analyses of growth behavior in mosquito cells was provided by the Institut Pasteur de Dakar, WHO Collaborating Center for arboviruses and viral hemorrhagic viruses (CRORA) in Senegal; this data is gathered in Table 3.

### 2.6. Data Analysis

Perl and R scripts were used to analyze codon usage data in this study, and are available from the authors upon request. In addition, the percentage of immunofluorescence, the logarithm of RNA copy number and the logarithm of viral titers are supposed to be normally distributed, and a Student’s test was used to analyze differences between virus strains.

## 3. Results

### 3.1. Temporal Trends in CAI Changes to Host Genes

**Figure 1:**
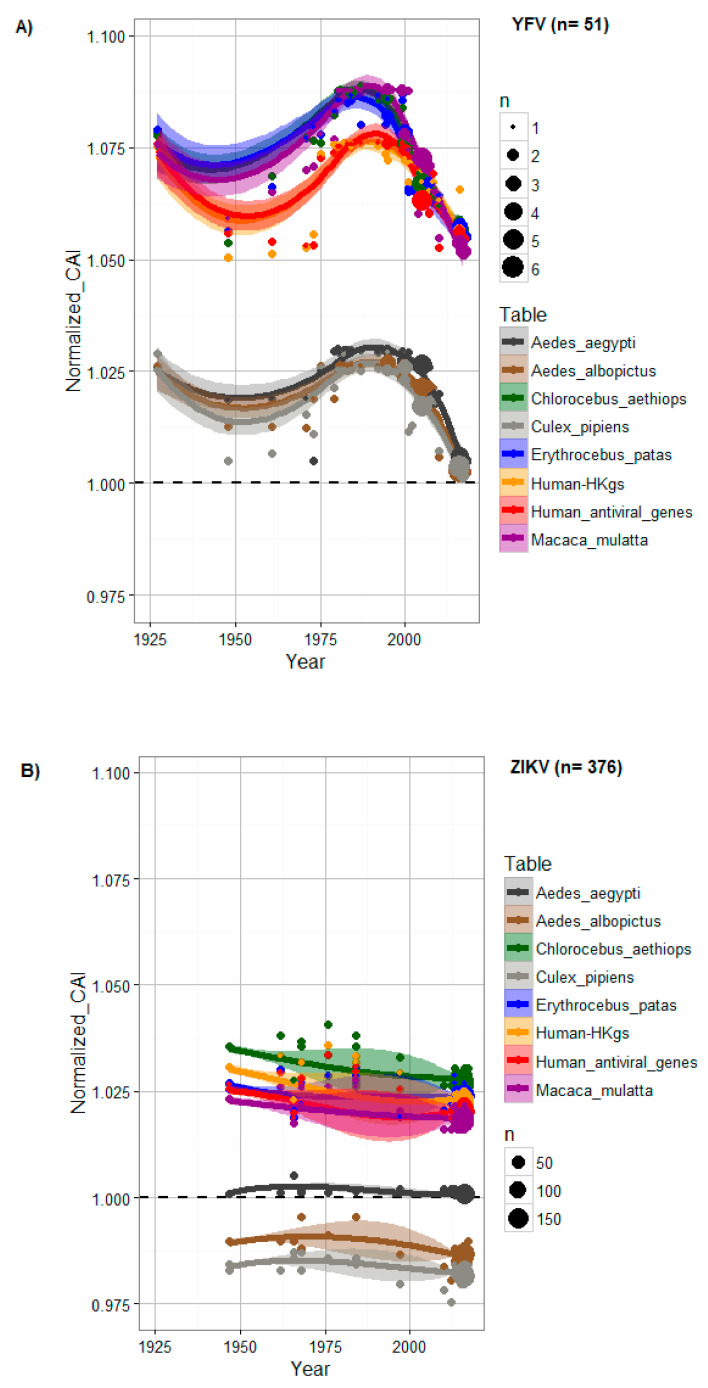
CAI changes to host genes over time for (**A**) YFV and (**B**) ZIKV. For each codon usage table, the CAI was normalized by length, percentage of GC and amino acid content for each dataset. The area of the plot points reflects the density of sequences at a specific time point (year). A CAI trend line above 1 (dashed black line) shows evidence of codon adaptation to the host. A trend line was generated using LOESS, a nonparametric regression method, with a 0.95 confidence interval (shaded areas). Data obtained from codon usage tables are colored in dark grey for *Aedes aegypti*, in brown for *Aedes albopictus*, in green for *Chlorocebus aethiops*, in grey for *Culex pipiens*, in blue for *Erythrocebus patas*, in orange for human housekeeping genes (HKgs), in red for highly expressed human antiviral genes (AVgs) and in purple for *Macaca mulatta*.

We explored possible changes in CAI over time for YFV and ZIKV using human, monkey and arthropod vector codon usage tables. Both had large numbers of available, serially stamped, complete polyprotein sequences, allowing us to investigate the temporal trends of ZIKV CAI, and to compare differences in the CAI of YFV strains (Figure 1).

YFV demonstrated codon adaptation for both vectors and mammalian hosts across all sampled strains, and the eight CAI trend lines were highly correlated (Spearman’s rank correlation test, *p*-values < 2.2 × 10^−16^, rho ranging from 0.988 to 0.999). YFV CAI was significantly higher for mammalian than that for vector codon usage (Wilcoxon rank sum test, *p*-values < 2.2 × 10^−16^). In addition, YFV CAI was significantly higher for monkeys than for human codon usage (Wilcoxon rank sum test, *p*-values < 0.00248), and could be associated with the important role of these monkey species in enzootic transmission of YFV. We also observed significant CAI differences between mosquito vectors with higher CAI for *Aedes aegypti* than for *Aedes albopictus* and *Culex pipiens* codon usage (Wilcoxon rank sum test, *p*-values < 0.0004), indirectly confirming *Aedes aegypti* as the main vector for YFV. No significant differences of YFV CAI were observed for HKgs and AVgs (Wilcoxon rank sum test, *p*-value = 0.82), or between monkey species (Wilcoxon rank sum test, *p*-values ranging from 0.461 to 0.560) (Figure 1A).

Similar to YFV, a positive correlation was observed in ZIKV codon adaptation to all eight trend lines (Spearman’s rank correlation test, *p*-values < 2.2 × 10^−16^, rho ranging from 0.953 to 0.996). However, ZIKV showed a higher CAI towards HKgs than to AVgs (Wilcoxon rank sum test, *p*-value < 2.2 × 10^−16^). This effect could have an important impact on the intrinsic biological characteristics of ZIKV during human infection, such as the level of immune response induction.

Across all eight codon usage tables, ZIKV had the highest CAI to *Chlorocebus aethiops* (green African monkey) codon usage (Wilcoxon rank sum test, *p*-value < 2.2 × 10^−16^). CAI values were significantly higher for *Aedes aegypti*, the main vector for ZIKV (Wilcoxon rank sum test, *p*-values < 2.2 × 10^−16^), and below 1 for *Aedes albopictus* and *Culex pipiens*, indicating no evidence for codon adaptation for these mosquito species (Figure 1B).

On average, ZIKV strains displayed 0.25–0.40 lower CAI values than the YFV strains for all eight codon usage tables tested. In addition, temporal trends have exhibited a variation in ZIKV population size during the last decade, while YFV showed a large population size before the 2000s (Figure 1A,B).

### 3.2. CAI Values of Host Genes

**Figure 2:**
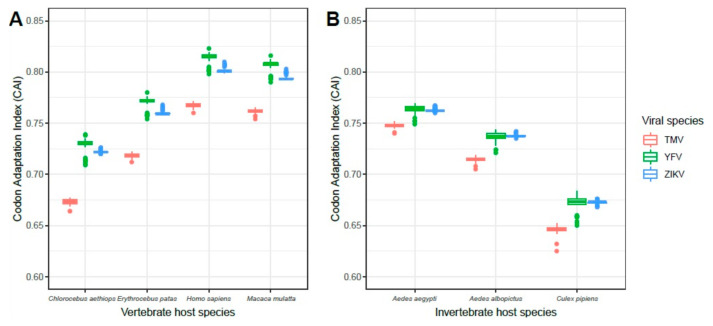
CAI value comparison between ZIKV and YFV to (**A**) vertebrate hosts and (**B**) invertebrate vectors. TMV was included as a negative control, since it is not expected to have a codon bias to animals. Data obtained for TMV, YFV and ZIKV are colored in red, green and blue, respectively.

The results of the calculation of the CAI values for different hosts, including vertebrates and invertebrates, indicated that ZIKV and YFV had a similar distribution of CAI values for invertebrate vectors and vertebrates, but that ZIKV had slightly lower CAI values than YFV. As expected, both had higher CAI values for all hosts when compared to TMV. Among vertebrate hosts, *Homo sapiens* presented the highest values, considering the distribution of CAI values for both arboviruses tested. For invertebrates, *Aedes aegypti*, followed by *Aedes albopictus*, which are considered to be important vectors for the transmission of arboviruses to vertebrates (Figure 2), had the highest CAI values.

**Figure 3:**
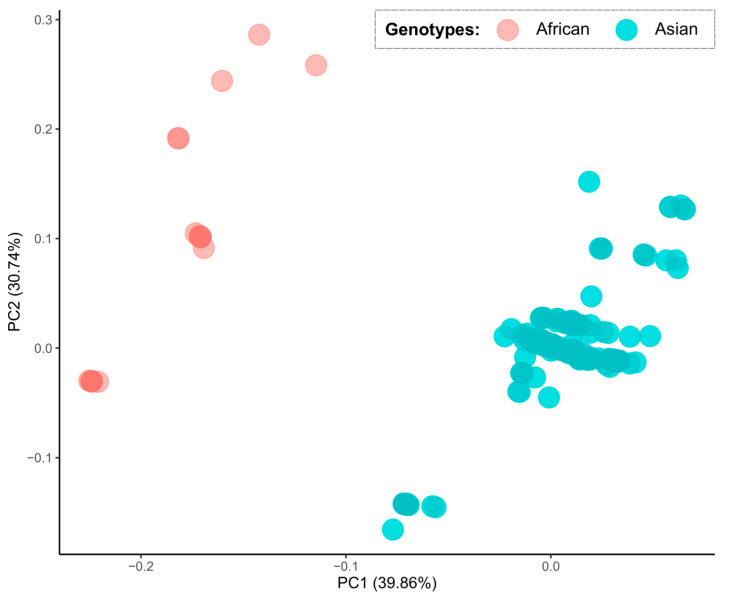
Principal component analysis comparing the CAI values to the *Homo sapiens* and to the *Aedes aegypti* mosquito, comparing between African (red dots) and Asian (blue dots) Zika virus genotypes.

**Figure 4:**
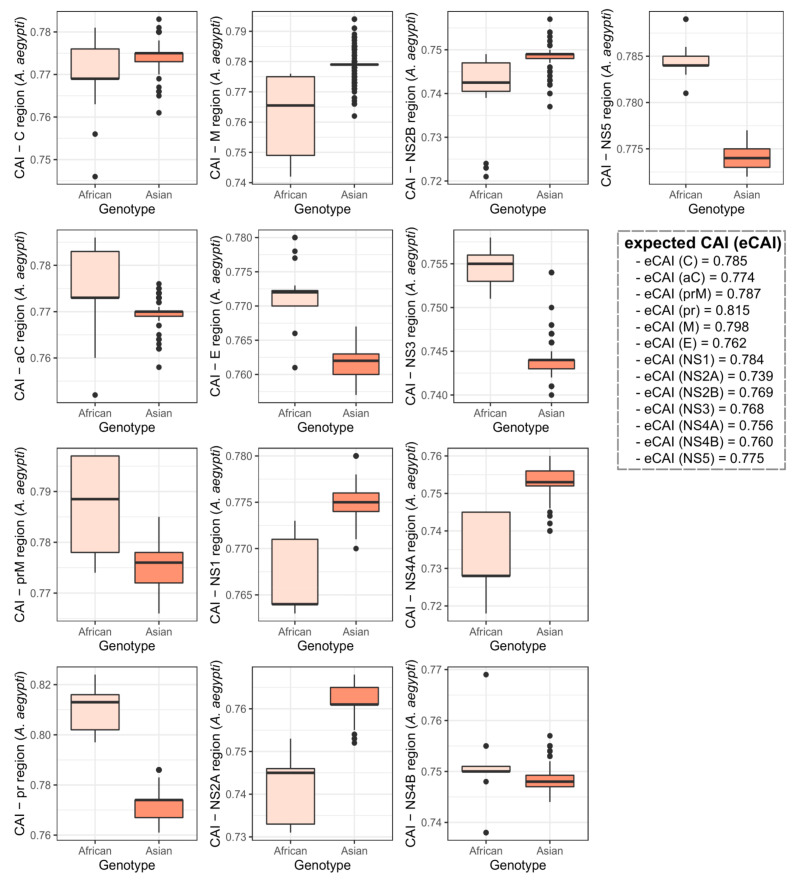
Comparison of the CAI values at the individual ZIKV gene/genomic region levels between the African and Asian ZIKV genotypes to the *Aedes aegypti* mosquito, the main urban ZIKV vector. Data obtained for the African and Asian ZIKV genotypes are colored in light orange and dark orange, respectively.

**Figure 5:**
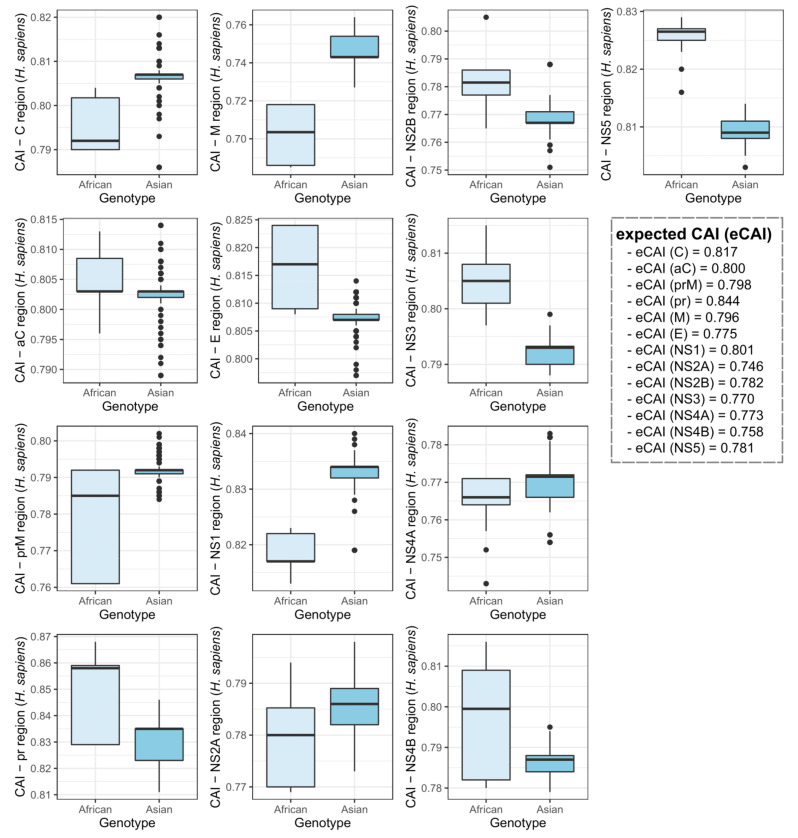
CAI values for the African and Asian ZIKV genotypes to *Homo sapiens* at the individual ZIKV gene/genomic region levels. Data obtained for the African and Asian ZIKV genotypes are colored in light blue and sky blue, respectively.

A principal component analysis on hosts with the highest CAI values (*H. sapiens* and *A. aegypti*) indicated a grouping of the two ZIKV genotypes in different groups. This suggested that each genotype may interact with distinct host species (Figure 3). A CAI analysis at the individual ZIKV gene level and/or genomic regions indicated clear differences between the two genotypes. The most evident distinction was observed on the ZIKV coding region for the NS1 protein, which had CAI values higher in the Asian compared to the African genotype for both human and mosquito (Figure 4 and Figure 5).

### 3.3. In Vitro Growth Kinetics of African ZIKV Strains

In order to understand the biology of the African genotype ZIKV in mosquito vector and vertebrate hosts, the replication kinetics of six African genotype ZIKV strains were determined in AP61 and Vero cells. Infection, viral proliferation and virulence in each cell type were measured by three different tests over a time period of 146 hpi.

**Figure 6:**
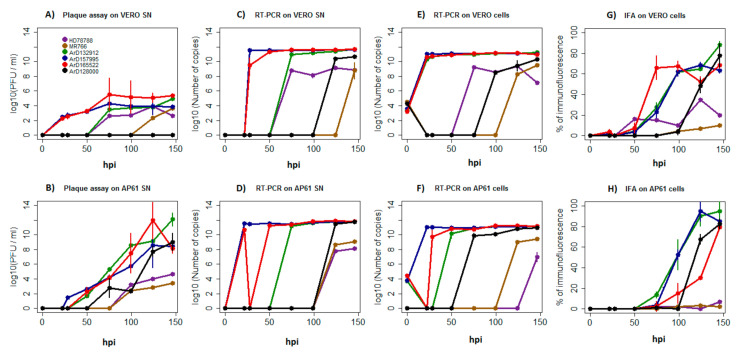
Growth kinetics of African Zika virus strains. The strain label is in reference to the strains in Table 2. Figure 6A–H show the number of infectious viral particles (**A** and **B**) (log10 of PFU/mL), the amount of viral RNA equivalents isolated from supernatants (**C** and **D**) and cell fractions (**E** and **F**) (log10 of RNA copy number), and the percentage (%) of immunofluorescence of viral antigen production (**G** and **H**) over a time period of 146 h post infection (hpi). The experiments were performed using the vertebrate cell line Vero (top row) and the mosquito cell line Ap61 (bottom row). Cells were infected with the human strain HD78788*^E^, the reference monkey strain MR766 and the mosquito strains ArD165522, ArD132912, ArD157995^*E/NS5^, ArD128000^*E/NS5^ colored in purple, brown, green, blue, red and black, respectively. The error bars indicate the range in values of three independent experiments performed using a multiplicity of infection (MOI) of 0.01.

We observed that African ZIKV strains had distinct growth dynamics in each cell line. In vertebrate cells, the strains ArD157995^*E/NS5^ and ArD165522 presented a significantly higher production of infectious particles than other African strains from 22 hpi to 50 hpi (*p*-values ranging from 4.25 × 10^−12^ to 0.0001). The strain ArD132912 and the human strain HD78788^*E^ showed a production of infectious particles from 75 hpi while the reference monkey strain MR766 released infectious particles very late, i.e., from 99 hpi. Strikingly, we did not detect any infectious particles for the strain ArD128000^*E/NS5^ isolated from a mosquito over a period of 146 hpi (*p*-values ranging from 9.26 × 10^−10^ to 4.79 × 10^−1^). However, no significant difference was found between other African ZIKV strains from 75 hpi to 146 hpi (Figure 6A). The highest replication after 146 hpi was recorded for strains ArD157995^*E/NS5^, ArD165522 and ArD132912, which showed similar growth dynamics. Significantly reduced replication efficiency was observed for the reference strain MR766, reduced replication for the human strain HD78788^*E^ and the mosquito strain ArD128000^*E/NS5^ (Figure 6C,E). The strains ArD157995^*E/NS5^, ArD165522, ArD132912 and ArD128000^*E/NS5^ showed a higher efficiency in the production of viral antigens, while the reference monkey strain MR766 and the human strain HD78788^*E^ were characterized by significant lower production of viral proteins at 124 hpi and 146 hpi (*p*-values ranging from 7.32 × 10^−15^ to 0.0001) (Figure 6G).

In mosquito cells, the mosquito strains ArD165522, ArD132912, ArD157995^*E/NS5^ and ArD128000^*E/NS5^ displayed higher infectious particle production, while the reference monkey strain MR766 and the human strain HD78788^*E^ released significantly reduced amounts of infectious particles from 124 hpi to 146 hpi (*p*-values ranging from 3.75 × 10^−8^ to 0.011) (Figure 6B). More efficient genome replication was also recorded for the mosquito strains ArD165522, ArD132912, ArD157995^*E/NS5^ and ArD128000^*E/NS5^, while the vertebrate strains MR766 and strain HD78788^*E^ replicated much less over 146 hpi (*p*-values ranging from 6.19 × 10^−9^ to 0.0001) (Figure 6D,F). Similar differences were also observed for viral antigens production, in which the reference monkey strain MR766 and the human strain HD78788^*E^ showed also lower significant rates than the other ZIKV strains from 124 hpi to 146 hpi (*p*-values ranging from 9.44 × 10^−7^ to 1.13 × 10^−1^) (Figure 6H).

Furthermore, cell line-specific culture differences were observed among African ZIKV strains. Despite quiet similar quantities of viral particles being released into the supernatants by each strain in both cell lines, African ZIKV strains produced up to 4 Log_10_ more infectious viral particles in mosquito cells than in vertebrate cells (Figure 6A,B); this indicates differing replication efficiencies in both cell lines or even incomplete replication (Figure 6A–D). For example, infectious particles for the strain ArD128000^*E/NS5^ were not detected in supernatants from the vertebrate cells over a time period of 146 hpi, while the titer of infectious particles (PFU/mL) increased significantly in mosquito cell supernatants (*p*-values ranging from 2.63 × 10^−12^ to 0.035) (Figure 6A,B).

Cell-specific replication differences were obvious for strain ArD165522, which showed a significant decrease at harvesting times 22 hpi and 28 hpi in cell fractions and supernatants from mosquito cells, respectively, while it presented high replication rates in the vertebrate cell line during the corresponding time points (*p*-values ranging from 3.75 × 10^−10^ to 0.027). In addition, the human strain HD78788^*E^ released fewer RNA copies into supernatants from mosquito cells over 146 hpi, while we found a significantly higher genome copy number in supernatants from vertebrate cells (*p*-values ranging from 9.26 × 10^−10^ to 4.79 × 10^−3^) (Figure 6C–F). Significantly lower profiles of viral antigen production were also observed for the human strain HD78788^*E^ and the monkey strain MR766 in both cell lines when compared to the ZIKV strains ArD165522, ArD132912 and ArD157995^*E/NS5^ (*p*-values ranging from 7.32 × 10^−15^ to 0.002) and the strain ArD128000^*E/NS5^, which showed a significant increase in viral antigen production in both cell lines from 99 hpi to 146 hpi (*p*-values ranging from 2.51 × 10^−11^ to 6.08 × 10^−4^) (Figure 6G,H).

In summary, the African ZIKV strains exhibited distinct growth characteristics in each cell line, and especially in the mosquito cell line. These differences seem to be associated with their host species origin, as the human strain HD78788^*E^ and the monkey strain MR766 were more cell-specific; both replicated more abundantly in vertebrate cells (Figure 6A–H), while the mosquito strains ArD165522, ArD132912, ArD157995^*E/NS5^ and ArD128000^*E/NS5^ displayed a more efficient growth in mosquito cells regarding the production of infectious particles (Figure 6A,B).

Among recombinant ZIKV strains, we observed different profiles. The E and NS5 recombinant strain ArD157995^*E/NS5^ replicated more efficiently in both vertebrate and mosquito cell lines. The E and NS5 recombinant strain ArD128000^*E/NS5^ exhibited an intermediate replication profile in both cell lines, while the E recombinant strain HD78788^*E^ presented much lower growth dynamics, particularly in mosquito cells (*p*-values ranging from 1.59 × 10^−13^ to 0.019) (Figure 6A–H). These different replication profiles may be associated with recombination in the NS5 protein or the simultaneous presence of recombination in both the E and NS5 proteins, since strains presenting these changes replicated more abundantly than the E recombinant strain HD78788^*E^. However, the replication levels of recombinant strains were not significantly distinct from those of nonrecombinant strains, considering the presence of profiles with more or less efficient replication in both groups.

Interestingly, the strains ArD165522, ArD132912 and ArD157995^*E/NS5^, presenting the N-glycosylation motif NDI at Asparagine 154 of the E protein, showed more efficient growth replication than strains ArD128000^*E/NS5^ and HD78788^*E^ with NDT motif or the strain MR766, which showed complete deletion of that motif (*p*-values ranging from 3.11 × 10^−10^ to 0.001). This effect was more marked in the vertebrate cell line, and showed that N-glycosylation on the E protein can play a major role in changes during growth of African ZIKV strains (Figure 6A–H).

**Figure 7:**
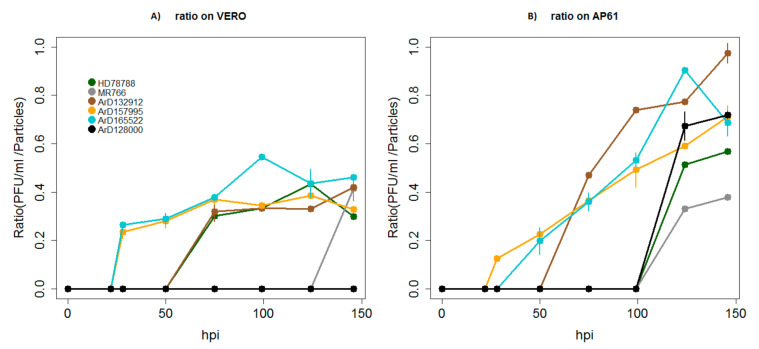
Ratios of the number of plaque forming units of African Zika virus divided by the total number of particles released in the supernatants (PFU/mL/Particles) in vertebrate cells Vero (A) and mosquito cells Ap61 (B) cell lines. The strain label is in reference to the strains in Table 2. Figure 4A,B shows the replication efficiency for each strain in each cell line (A and B) over a time period of 146 h post infection (hpi). Ratios for the human strain HD78788^*E^ are colored in green, the reference monkey strain MR766 is represented in dark gray line and the mosquito strains ArD165522, ArD132912, ArD157995^*E/NS5^ and ArD128000^*E/NS5^ are highlighted in brown, orange, light blue and black, respectively.

Finally, we estimated the replication efficiency by finding the ratios of the number of plaque forming units divided by the total number of particles released in the supernatants (PFU/mL/Particles) from each time point for each strain in each cell line. We found significant differences in replication efficiency with much higher and increasing ratios over 146 hpi observed in mosquito cells (*p*-values ranging from 6.32 × 10^−16^ to 0.0013) (Figure 7A,B). African ZIKV strains produced more infectious viral particles in mosquito cells than in vertebrate cells. This effect was very pronounced for the recombinant strain ArD128000^*E/NS5^, which produced no infectious particles at all in Vero cells over 146 hpi (Figure 7A,B). Reference monkey strain MR766 was shown to be the least efficient of all the ZIKV strains tested in both cell lines.

### 3.4. Replication Growth of African YFV

Since YFV and ZIKV share the same main vector, i.e., *Aedes aegyti*, their growth behaviors were compared in mosquito AP61 cells.

**Figure 8:**
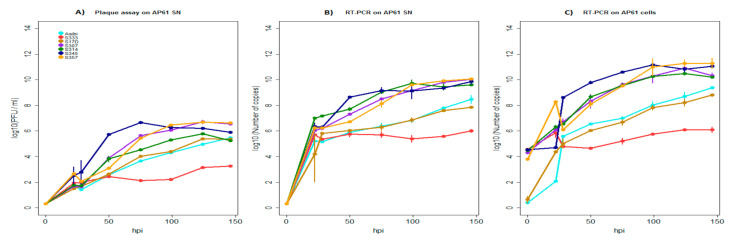
Replication growth kinetics of African Yellow Fever virus on mosquito cells (Ap61). Figure 8A–C show the number of infectious viral particles (**A**) (log10 of PFU/mL), the amount of viral genome copies quantified from supernatants (**B**) and cells (**C**) (Log10 of RNA copy number) over a time period of 146 h post infection (hpi). The strain label is in reference to the strains in Table 3. Data obtained with the reference wild-type strain Asibi are colored in light blue and the curves in red, brown, purple, green, dark blue and orange represent strains 333, 17D, 307, 314, 345 and 357, respectively. The error bars indicate the range in values of three independent experiments performed using a multiplicity of infection (MOI) of 0.01.

The growth dynamics of African YFV showed that strains 307, 357, 345 and 314 (belonging to lineages 1, 4, 5 and 6, respectively) exhibited high growth efficiency regarding the number of RNA copies and infectious particles produced in mosquito cells. The reference wild-type strain Asibi and the attenuated vaccine strain 17D replicated less efficiently, while strain 333 (lineage 3) displayed the lowest replication efficiency (Figure 8A–C).

In comparison to YFV strains, the growth of African ZIKV strains in mosquito cells was much slower, but with the exception of the human strain HD78788^*E^, all African ZIKV strains replicated more abundantly (from 2 to 6Log10) than YFV strains and produced significantly more infectious particles in cell supernatants (*p*-values ranging from 5.02 × 10^−6^ to 0.001) (Figure 6B–F and Figure 8A–C). These results suggest a high-titer virus production for the African genotype ZIKV in *Aedes* mosquito cells, which could play an important role in its natural maintenance.

## 4. Discussion

As seen for YFV and West Nile virus (WNV), first isolated in Africa, ZIKV is another flavivirus which spread from Africa to other continents [2,17]. The genetic diversity and broad host range of ZIKV [54] might have contributed to its extensive dissemination, and the A188V substitution in the NS1 protein of Asian ZIKV strain may be key to ZIKV spread from southeastern Asia across the Pacific islands to the Americas [55,56]. Its natural transmission cycle mainly involves monkeys, arboreal mosquitoes and, occasionally, humans.

The evolutionary adjustment of viruses to hosts through codon usage adaptation may reveal the role of host in natural maintenance of particular viruses.

Temporal analyses of CAI are informative in better identifying time points, which were marked by advantageous nonsynonymous changes in different hosts. Despite low CAI changes observed in its evolution, ZIKV shows slow evolutionary dynamics just like those of YFV [17,36,57,58]. Nevertheless, this slow evolution dynamic might be helpful for efficient replication of RNA viruses in the host, as more than one codon can be used for the same amino acid [56,59,60]. The significant lower CAI to AVgs than to HKgs could be linked to the ability of ZIKV to evade the immune system, in addition to inhibiting IFN production, as previously reported [61]. In addition, the higher CAI of ZIKV to *Chlorocebus aethiops* (green African monkey) is not surprising, because several monkey species have been identified as primary reservoirs of ZIKV [62], and therefore this monkey species could be involved in sylvatic ZIKV transmission in Africa. However, the recent detection of ZIKV in neotropical primates (*Sapajus libidinosus*) highlights the possibility that other host species could be involved in a sustained sylvatic cycle in the Americas [63], but perhaps also in Africa, and their role in ZIKV maintenance needs to be understood.

The abrupt variation observed in ZIKV population sizes over time could be correlated to the rapid increase of available sequencing data [64] and to the recent wide distribution of ZIKV from the Pacific Islands to the Americas [30,31,32,33]. The high NS1 CAI values of the Asian genotype for the human genome confirmed in this study were associated with the Asian genotype ZIKV for humans [65].

The lower CAI values obtained for the *Aedes aegypti* mosquito vector could explain the low competence for ZIKV for *Aedes aegypti* in the Americas [66] or the insignificant ZIKV transmission by *Aedes aegypti* in Africa, where it is transmitted by several other *Aedes spp.* mosquitoes, such as *Aedes vittatus* and *Aedes luteocephalus* [6]; this raises the question of the main *Aedes* vector for ZIKV in Africa.

Despite multiple isolations from mosquito populations and serological evidence of ZIKV infection in Africa, a major outbreak in humans attributed to the African genotype ZIKV has not yet been documented. Codon bias is a common mechanism across the genomes of several organisms and plays a major role in their evolution. The significant ZIKV adaptation to the genome of monkeys could lead to possible spillover of the African genotype ZIKV to other vertebrates such as humans, as exhibited by the high CAI for the human genome, which is actually already higher than the CAI of Asian genotype ZIKV. This clearly shows that the African genotype ZIKV also has the potential to efficiently replicate in humans. This is particularly true for the CAI for the E and NS5 proteins [65], which have a major role in flaviviruses replication and virulence [17].

African genotype ZIKV presents two major clades, which have previously experienced probable recombination and glycosylation mechanisms in their natural cycle [17,28]. To better understand its biology in mosquito vectors and vertebrate hosts and assess the effect of these evolutionary mechanisms on its fitness, the in vitro phenotypical behavior of African ZIKV strains was analyzed over a 146 hpi period. Thus, infection, viral proliferation and virulence in each cell type were assessed using RT-qPCR of the lysed cell fraction to measure genome replication, RT-qPCR of the supernatant fraction to detect genome replication dynamics (i.e., total number of particle released), plaque assays to determine the amount of infectious viral particles (PFU/mL) from the supernatant fraction, and immunofluorescence staining of the cells to estimate efficiency in viral antigen production.

While some differences were previously reported between African and Asian ZIKV strains [66,67,68,69,70], our study focuses on phenotypic differences between African ZIKV strains by characterizing their growth behaviors in two distinct cell lines. The observed high-titer virus production in mosquito cells and significantly reduced levels in vertebrate cells may reflect ZIKV maintenance in African transmission cycles.

The replication levels of African ZIKV strains isolated from mosquitoes in the vertebrate cell line confirms that these isolates are able to efficiently infect and replicate in vertebrate cells, as, for example, reported for neuronal cells [70,71]. However, the lower replication efficiency in the vertebrate cell line might contribute to the inability of African genotype ZIKV to cause severe pathogenicity in vertebrates such as humans.

Indeed, the African genotype ZIKV has been shown to induce less pathogenicity in human cells than the epidemic Asian genotype ZIKV, which shows long-term persistence, more consistent with clinical manifestations [72,73,74]. The reduced pathogenicity exhibited by the African genotype ZIKV in human cells in vitro was correlated with the induction of early cell death and a strong antiviral response [41,48,70,71,75], which explains the low amount of infectious viral particles produced in vertebrate cells in our study.

African ZIKV strains are more virulent in mosquito cells [76]. This observation is supported by our assessment of replication efficiency in a mosquito cell line (PFU/mL/Particles), shown in Figure 4.

However, cell-specific phenotypic differences observed between African ZIKV strains used in our study might be related to the intrinsic properties of the African ZIKV strains, their species origin or differential susceptibility to ZIKV infection for the cell lines used in our study [77,78,79]. Indeed, mosquito strains ArD157995^*E/NS5^, ArD165522, ArD132912 and ArD128000^*E/NS5^ replicated more efficiently in the mosquito cell line, while the human strain HD78788^*E^ and the monkey strain MR766 showed higher replication in the vertebrate cell line. It is of note that the prototype ancestral ZIKV strain MR766 replicated significantly more slowly. At least three MR766 strains exist with genetic differences [80]. Our results indicate that strain MR766, which is used in the majority of ZIKV research studies, cannot be considered as a reference, as it shows quite atypical ZIKV replication in both vertebrate and mosquito cells.

Like other RNA viruses cycling between arthropod vector and mammal hosts, it was previously found that the majority of codon sites in E and NS5 proteins of ZIKV undergo purifying selection [17,51,81]. Nevertheless, some significant diversifying sites exist in the genome of the African genotype ZIKV, such as recombination sites in E and NS5 proteins and the N-glycosylation motif (NDT) at codon positions 154–156 of the E protein, which plays a crucial role in the replication and virulence of flaviviruses [17,51,52,82,83]. Although recombination could not be associated with one specific replication profile of African ZIKV strains in our study, the significant replication difference between strains ArD128000^*E/NS5^ and HD78788^*E^, particularly in mosquito cells, points to the detrimental impact of recombination in the NS5 protein on virulence and viral infectivity. The effect of recombination on replication efficiency and virulence of ZIKV has long been suspected based on phylogenetic studies [17,84,85]. The recombinant strain ArD128000^*E/NS5^, which replicated efficiently in mosquito cells but exhibited no production of infectious particles in the vertebrate cell line over 146 hpi, indicated that recombination has an effect on host tropism.

In contrast to a previous in vivo study demonstrating that complete deletion—as observed in most of the African ZIKV strains—or single amino acid substitution in the NDT motif cause less infectivity [86], our study shows that the NDI motif at codon position 156 of the E protein is an important determinant of the virulence and replication of African ZIKV strains in vertebrate cells.

The recombination and N-glycosylation may lead to potential future adaptation and pathogenicity of the African genotype ZIKV, and the biological and genetic characteristics of new isolates need to be continuously monitored.

However, considering functional differences between in vitro and in vivo systems in terms of physical barriers and antiviral responses, the fitness of African ZIKV strains with a NDI motif on the E protein should be studied in vivo, since many other amino acid substitutions contributing to ZIKV pathogenicity have been previously identified [87,88,89]. Enhanced infectivity of ZIKV in the *Aedes aegypti* mosquito vector has been correlated with the signature A188V mutation in NS1, which potentially facilitates transmission, and therefore, contributed to the spread of the virus from Asia to the Americas [88,89].

In contrast to faster in vitro replication of African YFV in mosquito cells, the African ZIKV strains exhibited more efficient replication, yielding more infectious particles. In contrast to YFV, which has a long history of human infection in Africa [42,90], the African genotype ZIKV currently appears to be maintained through a sylvatic cycle, and human infections still appear to be rare. However, our own serological data from active surveillance of arboviruses in Senegal indicate that asymptomatic ZIKV infections may be more widespread than assumed [91]. If confirmed, asymptomatic or unnoticed infection could be an alternative explanation for the higher CAI values for the human genome of the African genotype ZIKV.

In summary African genotype ZIKV isolates replicate more efficiently in mosquito cells than in vertebrate cells. Their CAI indices, however, indicate a higher adaptation to vertebrates than observed for Asian genotype ZIKV, which may reflect efficient sylvatic transmission cycles but possibly also asymptomatic transmission in a peri-urban cycle. An analysis of the growth characteristics of individual African genotype ZIKV strains revealed that strain MR766 is not suitable as a reference strain, while recombinant strain ArD128000^*E/NS5^ may be a good model for use studies on the mechanism of host tropism.

## Figures and Tables

**Table 1:** Codon frequency tables for the different hosts considered in the analysis.

Host Organism	Host	Source	Date of Acquisition	Number of Coding Sequences (CDS)	Number of Codons	Taken from
*Homo sapiens*	Vertebrate	CoCoPUTs	11 June 2019	119846	78581299	RefSeq
*Macaca mulatta*	Vertebrate	CoCoPUTs	20 July 2020	67214	46960666	RefSeq
*Erythrocebus patas*	Vertebrate	CoCoPUTs	20 July 2020	165	47999	GenBank
*Chlorocebus aethiops*	Vertebrate	CoCoPUTs	20 July 2020	621	183383	GenBank
*Aedes aegypti*	Invertebrate	CoCoPUTs	29 May 2019	28043	20013993	RefSeq
*Aedes albopictus*	Invertebrate	CoCoPUTs	20 July 2020	37335	22045256	RefSeq
*Culex pipiens*	Invertebrate	CoCoPUTs	20 July 2020	248	52907	GenBank

**Table 2:** Zika virus strains used in this study.

Strains	Species	Place of Isolation	Year of Isolation	African Sub-Clades	Number of Passages	Titers of Initial Stocks (PFU/mL)	N-glycosylation Motif (Ng)	Acc. Numbers
ArD128000^*^^E/NS5^	*Aedes luteocephalus*	Senegal	1997	Nigerian	4	2.5 × 10^12^	N-D-T	KF383117
ArD132912	*Aedes dalzieli*	Senegal	1998	Nigerian	3	1.75 × 10^7^	N-D-I	KF383021, KF383096
ArD157995^*E/NS5^	*Aedes dalzieli*	Senegal	2001	Nigerian	4	1.75 × 10^5^	N-D-I	KF383118
ArD165522	*Aedes vitt* *atus*	Senegal	2002	Nigerian	3	3.5 × 10^7^	N-D-I	KF383029, KF383090
MR766	*Macaca mulatta*	Uganda	1947	Ugandan	5	1.5 × 10^9^	-----	KX421193
HD78788^*E^	*Homo sapiens*	Senegal	1991	Nigerian	5	1.25 × 10^8^	N-D-T	KF383039, KF383084

^*^^E^: Recombinant strain, breakpoints on Envelope (E); ^*E/NS5^: Recombinant strain, breakpoints on Envelope (E) and polymerase (NS5) proteins (30); Ng: N-glycosylation site at Asn-154 of the Envelope protein (Asn-X-Thr) (25, 30).; Asn: Asparagine (N), Asp: Asparatate (D), Ile: Isoleucine (I), Thr: Threonine (T), “-----”:Complete deletion.

**Table 3:** Yellow fever strains used in analyses of growth behavior.

Strains	Species	Isolates	Place of Isolation	Year of Isolation	Lineage	Acc. Numbers
333	*Aedes aegypti*	ArD 114896	Senegal	1995	3	JX898871
307	*Aedes africanus*	DakArAmt7	Côte d’Ivoire	1973	1	JX898869
357	*Aedes furcifer*	ArD 156468	Senegal	2001	4	JX898876
345	*Aedes furcifer*	ArD 149214	Senegal	2000	5	JX898873
314	*Aedes furcifer*	ArD 121040	Senegal	1996	6	JX898870
Asibi	*Homo sapiens*		Ghana	1927		KF769016
17D	*Homo sapiens*	17D RKI #142/94/1				JN628279
